# Effects of Age and Larval Nutrition on Phenotypic Expression of Insecticide-Resistance in *Anopheles* Mosquitoes

**DOI:** 10.1371/journal.pone.0058322

**Published:** 2013-03-06

**Authors:** Katarzyna Kulma, Adam Saddler, Jacob C. Koella

**Affiliations:** 1 Department of Animal Ecology, Uppsala University, Uppsala, Sweden; 2 Division of Biology, Imperial College London, Ascot, United Kingdom; National Institute for Communicable Diseases/NHLS, South Africa

## Abstract

Insecticide-resistance threatens the control of mosquito-borne diseases like malaria or dengue fever. To ensure sustainable vector control we need a full understanding of the factors driving the evolution of resistance. We test the hypothesis that the expression of insecticide-resistance depends on the available resources by rearing genetically DDT-resistant and sensitive larvae of *Anopheles* mosquitoes at three diet regimes, which correspond to 40%, 70% and 100% of the normal diet and exposing the adult females to DDT 5, 10 and 15 days after emergence. In both colonies post-exposure survival decreased with age at exposure. Additionally, the food levels and DDT-resistance were positively correlated in both colonies, although only in the DDT-resistant one was this relationship statistically significant. The impact of larval diet was smaller than the effect of age at exposure. We discuss our results and explain the implication of this study to resistance monitoring for public health and vector management.

## Introduction

The evolution of insecticide-resistance threatens the control of mosquito-borne diseases like malaria or dengue fever [1]. With a view to manage the problem of resistance, research has led to considerable knowledge about the molecular mechanisms of resistance and the physiological routes leading to insecticide-resistance [2].

However, to ensure sustainable and efficient vector control we need a full understanding not only of the genetic and physiological basis of resistance, but also of the non-genetic factors that influence the response to insecticide and the expression of resistance. Some of these environmental and demographic factors have started to be understood.

First, sensitivity to insecticides increases as mosquitoes age [3]–[5]. This may be at least partly explained by an age-related decline in the expression of insecticide detoxification genes [4] (but see [3], [6] ). Second, parasite infections can modify the way genetically resistant mosquitoes respond to insecticides. For example, infection by entomopathogenic fungi or microsporidian parasites partially restores the mosquitoes’ sensitivity to insecticides [7], [8], and infection with an insecticide-degrading bacterial symbiont establishes insecticide-resistance in pest insects [9]. Third, the temperature in which mosquitoes are exposed to insecticides can affect their resistance, so that warmer conditions lead to higher mortality [10]. Finally, some studies suggest that providing mosquitoes with a blood meal may decrease their sensitivity to insecticides [3], [4].

Yet, it is still unclear whether and how available resources modify the expression of insecticide-resistance. It has been recently shown that metabolic resistance to insecticides uses resources essential for development [11], [12]: mosquitoes resistant to insecticides store fewer lipids, sugars and energetic reserves than sensitive ones, implying that the expression of the resistance is resource-dependent. [12]. We therefore expect that resource availability limits the expression of resistance.

To test this hypothesis, we reared DDT-resistant and sensitive larvae of *Anopheles gambiae* mosquitoes on three different feeding regimes, constituting 40%, 70% and 100% of their standard diet. Subsequently, we measured their resistance by exposing them to DDT at different ages and recording their survival 24 hours after the exposure. We predicted that the post-exposure survival would decrease with age and would be lower for the badly nourished mosquitoes than for the well-fed ones. We also expected that the negative effects of low diet would be stronger in the resistant colony than in sensitive mosquitoes because of the resource demands related to insecticide resistance.

## Materials and Methods

### Feeding Regime

We used two colonies of *Anopheles gambiae* mosquitoes: the DDT-resistant ZAN/U colony with increased metabolism of the insecticide, catalyzed by members of the glutathione S-transferees enzyme family [13] and the DDT-sensitive Kisumu colony from western Kenya [14]. Two-day old larvae were transferred to 12-well plates and reared individually in 3 ml of de-ionized water. For each colony we reared 480 mosquitoes at each of three feeding regimes: 100% (high), 70% (medium) or 40% (low) of the standard amount of TetraMin® Baby fish food (Table 1). Given food quantities were administered to each well in 100 uL of de-ionized water (which partially compensates for evaporative loss). Emerged females were moved to plastic cups and supplied with cotton balls moistened with saturated 10% sugar solution, males were discarded.

**Table 1:** Daily amounts of food (in µg) for the three different diet levels.

Days after hatch	LOW	MEDIUM	HIGH
**1**	0.016	0.028	0.04
**2**	0.024	0.042	0.06
**3**	0.032	0.056	0.08
**4**	0.064	0.112	0.16
**5**	0.128	0.224	0.32
**6 and later**	0.240	0.420	0.60

### Insecticide Exposures

The resistance of mosquitoes was measured with the standard World Health Organization test-kit according to WHO guidelines [15]. 50 adult females from each feeding regime and colony were exposed to insecticide at each of three ages: 5, 10 or 15 days after emergence. They were individually exposed to DDT-treated filter paper (4%) for 100 minutes (resistant ZAN/U colony) or 40 minutes (sensitive Kisumu colony). We based the exposure times on earlier experiments, so that we could expect about half of the to die within 24 hours of exposure when they were 5 days old. After exposure the mosquitoes were moved back into insecticide-free plastic cups and survival was recorded 24 h later. Mosquito rearing and insecticide exposures were carried out at a temperature of 26 (+/−1)°C and 70 (+/−5) % relative humidity with a 12 h: 12 h light/dark cycle. Subsequently, mosquitoes were individually moved to eppendorf tubes and frozen. We removed their wings, fixed them onto glass slides, scanned and measured from the tip (excluding the fringe) to the distal end of the allula using ImageJ software (http://rsb.info.nih.gov/ij/). Where both wings were available, we took their mean length as a measure of mosquito size.

### Statistical Analysis

**Figure 1:**
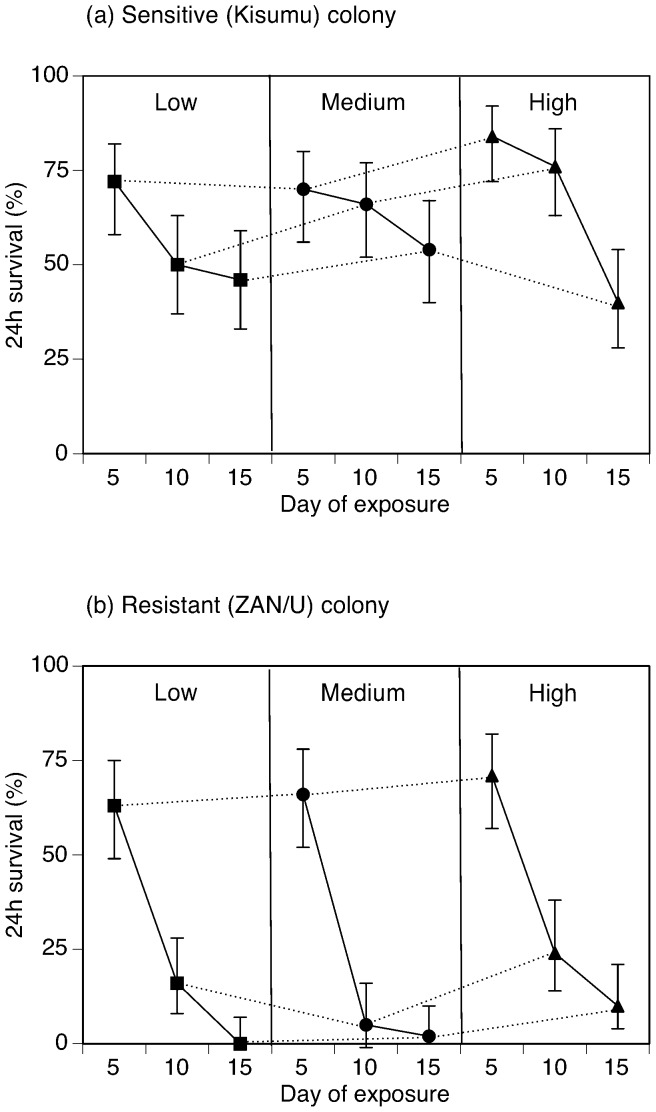
Survival 24 hours after exposure to DDT in (a) Kisumu (sensitive) and (b) ZAN/U (resistant) mosquitoes. In both panels survival is shown as a function of age at exposure. The error bars show the 95% confidence intervals. Dotted lines link mosquitoes from different treatment groups exposed at the same age. Note that the time of exposure differed between the two colonies: The Kisumu mosquitoes were exposed for 40 minutes; the ZAN/U mosquitoes were exposed for 100 minutes.

The mosquitoes emerged over a period of 5 days (ZAN/U; 9 to 13 days after hatching) and 3 days (Kisumu; 9 to 11 days after hatching), so we grouped mosquitoes not only by treatment (food regime and age at exposure), but also by age at emergence. The number of mosquitoes that survived the exposure in each group (food regime, age at exposure, age at emergence) was analysed with a binomial GLM with logit link, with a correction for over-dispersion if necessary. As age at emergence determined the date of exposure (so that age at exposure could be fixed), it was considered a nominal factor. As age at exposure had a close to linear effect (Figure 1), it was considered a continuous factor; feeding regime was considered an ordinal factor. The analysis included only the interaction between age at exposure and feeding regime, as including interactions with age at emergence would have led to a very unbalanced analysis. As the treatment of the two colonies differed, we analysed the data of the resistant and sensitive colony separately.

The analyses were carried out with the statistical package JMP 8.0.2 (SAS Institute, Cary, NC).

## Results

**Figure 2:**
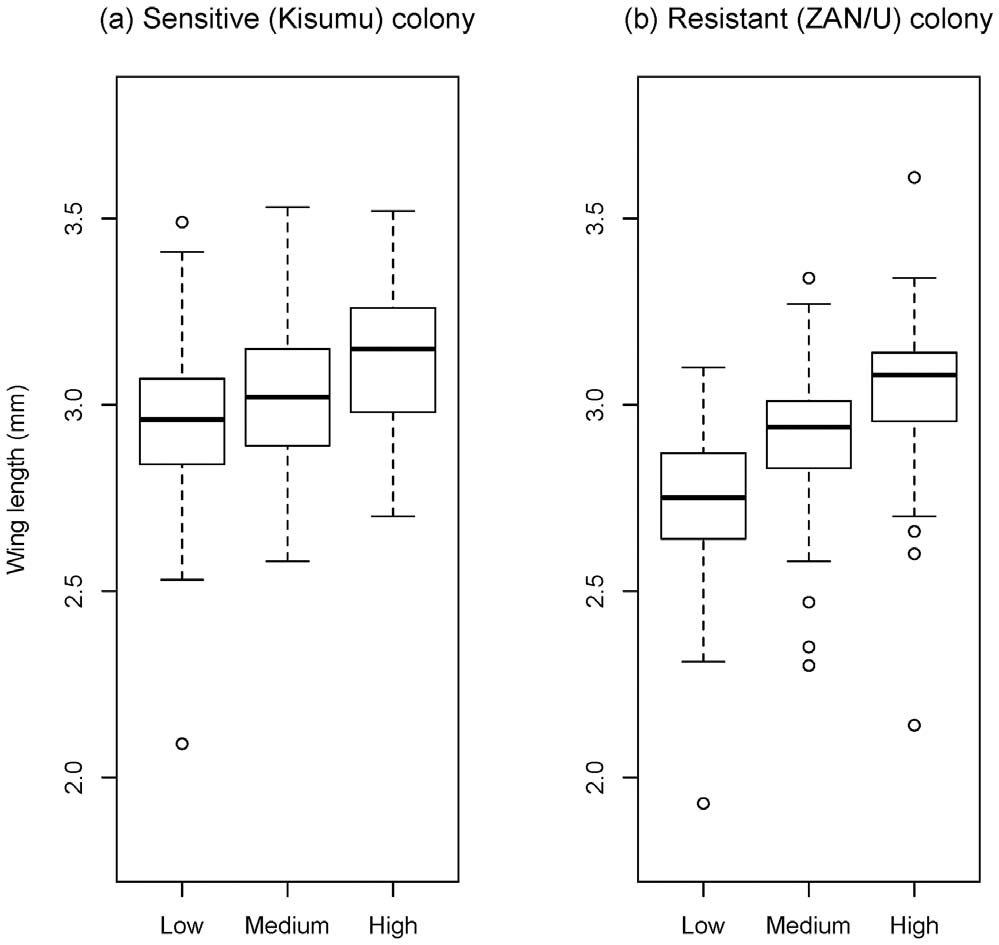
Boxplot of wing length by colony and diet. A positive correlation between diet and wing length is observed in both: a) Kisumu and b) ZAN/U colonies. Thick horizontal lines represent median, bottom and upper edges of the boxes first and third quartiles, whiskers demonstrate minimum and maximum values.

In sensitive and DDT-resistant mosquitoes decreasing larval diet delayed emergence (sensitive: chi-square = 565.0, d.f = 4, p<0.001; resistant: chi-square 547.8, d.f = 8, p<0.001) and decreased adult wing length (sensitive: F = 33.95, d.f. = 1,308, p<0.001; ZAN/U F = 129.97, d.f. = 1,286, p<0.001; Figure 2). In both colonies, age at exposure had a greater influence on survival than feeding regime (Figure 1). Age at emergence had only little effect on survival (Table 2). For the sensitive Kisumu mosquitoes, later exposure and less larval food decreased survival (Table 2). The effect of the feeding regime differed among the three ages at exposure; thus the effect of age at exposure was greatest in the best-fed mosquitoes, and increasing larval food increased resistance only in the mosquitoes exposed 5 or 10 days after emergence (Figure 1).

**Table 2:** GLM analysis of 24 hours post-exposure survival in a) resistant and b) sensitive colonies of *Anopheles gambiae* mosquitoes.

Factor	df	Χ^2^	P
**(a) resistant ZAN/U**			
Age at emergence	4	5.49	0.241
Age at exposure	1	68.01	**<0.001**
Food regime	2	3.63	0.163
Food* Age at exposure	2	4.38	0.112
**(b) sensitive Kisumu**			
Age at emergence	2	4.95	0.084
Age at exposure	1	10.72	**0.001**
Food regime	2	10.50	**0.005**
Food* Age at exposure	2	8.65	**0.013**

The pattern in the resistant ZAN/U mosquitoes was similar but stronger, with survival after exposure decreasing with age (Table 2, Figure 1). There was, however, little difference between the food regimes and no significant interaction between feeding regime and age at exposure (Table 2, Figure 1).

## Discussion

In our study, the quantity of food available to larvae had an effect on the expression of insecticide-resistance. However, its effect was small and was apparent only in one of our two colonies and if the mosquitoes were exposed fairly early after emergence. Furthermore, corroborating other studies [3]–[5], in both colonies post-exposure survival decreased with the age at exposure to DDT.

As insecticide-resistance often [11], [12], [16], but not always [17] has energetic and fitness costs, we had expected that our feeding regime (which had a strong effect on adult size (Figure 2) and thus, presumably on adult condition and level of stored resources [16], [17]) would affect the mosquitoes’ survival of the insecticide. However, there was only a small effect of larval nutrition on resistance. One reason may be due to the importance of resistance for the fitness of mosquitoes, so that allocation to resistance may be maintained even in face of low resource availability. Alternatively, the underfed larvae may have compensated for their low energy reserves by feeding more in the adult stage [18]. Finally, the cost of resisting the insecticide (which has not been measured for ZAN/U mosquitoes) may be minimal in our mosquitoes.

As the resistance mechanism requires increased production of metabolic enzymes and therefore presumably uses more resources, we had expected that ZAN/U mosquitoes would be more sensitive to food deprivation than Kisumu ones. We observed, however, the opposite: whereas the resistance of ZAN/U was not affected by larval food, Kisumu mosquitoes were less resistant if fed less as larvae. This suggests that the mutations of the detoxification genes in our resistant colony are less sensitive to environmental variation than the more general mechanisms that help to survive exposure to insecticides. Note, however, that any difference between ZAN/U and Kisumu must be interpreted with caution as their genetic backgrounds differ by more than just the site of resistance [19].

A significant interaction was observed between diet and age of exposure in the sensitive Kisumu colony. This is mainly a reflection of the survival in one treatment: high diet mosquitoes exposed 15 days after emergence (Figure 1). Without this treatment survival decreased with decreasing diet level. We therefore believe that the interaction may be an artefact of experimental error or other external interference (e.g. higher bacterial load due to higher food levels).

Our results confirm the importance of mosquito age in expression of insecticide resistance. We argue that in order to compare the level of resistance between different studies or sites, mosquito age needs to be considered. We additionally show that the age-related decline in resistance differs between the colonies and can be affected by larval food availability. Although the effect of larval diet was not as strong as the effect of age, our results emphasize that considering environmental variation is important to understand the expression of insecticide resistance. Such information would greatly benefit our understanding of evolution of resistance and could advice strategies for vector control initiatives.
